# *Cryptococcus gattii* alters immunostimulatory potential in response to the environment

**DOI:** 10.1371/journal.pone.0220989

**Published:** 2019-08-09

**Authors:** Keigo Ueno, Yoshiko Otani, Nao Yanagihara, Takumi Nakamura, Kiminori Shimizu, Satoshi Yamagoe, Yoshitsugu Miyazaki

**Affiliations:** 1 Department of Chemotherapy and Mycoses, National Institute of Infectious Diseases, Toyama, Shinjuku-ku, Tokyo, Japan; 2 Department of Biological Science and Technology, Faculty of Industrial Science and Technology, Tokyo University of Science, Niijuku, Katsushika-ku, Tokyo, Japan; Wadsworth Center, UNITED STATES

## Abstract

*Cryptococcus gattii* is a capsular fungal pathogen, which causes life-threatening cryptococcosis in immunocompetent individuals. This emerging pathogen is less likely to be recognized by innate immunity compared to traditional *Cryptococcus neoformans* strains. Previous studies indicate that C-type lectin receptors (CLRs), including dectin-1 and dectin-2, play a role in recognizing cryptococcal cells; however, it remains to be elucidated whether the receptors physically associate with *C*. *gattii* yeast cell surfaces. Based on the previous findings, we hypothesized that culture conditions influence the expression or exposure of CLR ligands on *C*. *gattii*. Therefore, in the present study, we first investigated the culture conditions that induce exposure of CLR ligands on *C*. *gattii* yeast cells via the binding assay using recombinant fusion proteins of mouse CLR and IgG Fc, Fc dectin-1 and Fc dectin-2. Common fungal culture media, such as yeast extract–peptone–dextrose (YPD) broth, Sabouraud broth, and potato dextrose agar, did not induce the exposure of dectin-1 ligands, including β-1,3-glucan, on both capsular and acapsular *C*. *gattii* strains, in contrast to Fc dectin-1 and Fc dectin-2 bound to *C*. *gattii* cells growing in the conventional synthetic dextrose (SD) medium [may also be referred to as a yeast nitrogen base with glucose medium]. The medium also induced the exposure of dectin-1 ligands on *C*. *neoformans*, whereas all tested media induced dectin-1 and dectin-2 ligands in a control fungus *Candida albicans*. Notably, *C*. *gattii* did not expose dectin-1 ligands in SD medium supplemented with yeast extract or neutral buffer. In addition, compared to YPD medium-induced *C*. *gattii*, SD medium-induced *C*. *gattii* more efficiently induced the phosphorylation of Syk, Akt, and Erk1/2 in murine dendritic cells (DCs). Afterwards, the cells were considerably engulfed by DCs and remarkably induced DCs to secrete the inflammatory cytokines. Overall, the findings suggest that *C*. *gattii* alters its immunostimulatory potential in response to the environment.

## Introduction

*Cryptococcus gattii* is an encapsulated fungal pathogen which infects to immunocompetent individuals and causes cryptococcosis. Following a *C*. *gattii* infection outbreak in North America since 1999, several highly virulent *C*. *gattii* strains, including R265 and JP02, have been isolated globally, and the relationship between their antigenic potential and their virulence has been investigated [1–4].

We have previously demonstrated that *C*. *gattii*-encapsulated strains, R265 and PNG18, are not engulfed by dendritic cells (DCs) and neutrophils without opsonization [5,6], and that the strains do not induce the secretion of the inflammatory cytokines, such as IL-12p40 and TNFα, by DCs [5]. In contrast to encapsulated wild type strains, capsular strain CAP60Δ strongly stimulated DCs to produce inflammatory cytokines and the costimulatory molecules and were efficiently phagocytized by DCs [5]. *C*. *gattii* is also less likely to be recognized compared to *Cryptococcus neoformans* under *in vivo* situations because *C*. *gattii* induces lower cytokines production and leukocyte recruitment in the lungs compared to *C*. *neoformans* [2–4]. The findings above suggest that *C*. *gattii* wild type strains have lower antigenic potential *in vitro* and *in vivo*.

In general, innate immune cells, such as DCs, recognize fungal pathogens via pattern recognition receptors (PRRs), including C-type lectin receptors (CLRs), toll-like receptors (TLRs), nucleotide-binding oligomerization domain (NOD)-like receptors (NLRs), and retinoic acid-inducible gene-I (RIG-I)-like receptors (RLRs)[7,8]. The representative CLRs are dectin-1 and dectin-2, which physically bind to fungal cell wall components β-1,3-glucan and α-mannan, respectively [9–12]. Such PRR ligands are referred to as pathogen-associated molecular patterns (PAMPs). The PAMPs induce the phosphorylation of Syk, Akt, and Erk1/2 in innate immune cells, including DCs via CLRs [13] and then stimulate the production of inflammatory cytokines by DCs [14]. Phagocytes also recognize the PAMPs, which facilitates the engulfing of fungal cells [14].

Several studies have suggested interactions between dectin-1 and cryptococcal cells by using dectin-1 deficient phagocytes [15,16], a dectin-1 expressing cell line [17], and a dectin-1 antagonist [18]. However, physical association has not been demonstrated between dectin-1 and yeast cells of cryptococcal cells. A recombinant fusion protein of mouse dectin-1 and IgG Fc (Fc dectin-1) has been used to investigate the recognition pattern of mouse dectin-1 [19]. A previous study has demonstrated that Fc dectin-1 binds to *C*. *neoformans* spores but not to yeast cells [20], whereas another study reported contradictory findings where anti-β-glucan antibodies bind to the yeast cells of capsular and acapsular *C*. *neoformans* [21].

A similar contradiction was also observed in the case of dectin-2. Compared to wild type, dectin-2 deficient macrophages and DCs engulfed *C*. *neoformans* cells less efficiently and produced lower amounts of inflammatory cytokines following cryptococcal stimulation [15,22]. In addition, it has been demonstrated that cryptococcal cell wall mannoprotein MP98 as well as *Blastomyces dermatitidis* glycoprotein Bl-Eng2, activate B3Z dectin-2 reporter cells and induce the secretion of inflammatory cytokine Interleukin (IL)-6 in DCs but not in dectin-2 deficient DCs [23,24]. However, Fc dectin-2 does not bind to cryptococcal cells, and intact cryptococcal cells do not activate dectin-2-NFAP-GFP reporter cells [12,22].

Because different studies have adopted varied culture conditions for cryptococcal cells, we infer that the different culture conditions can be the reason for the inconsistent observations described above. Although cryptococcal cells possess β-1,3-glucan and α-mannan in the steady state cell walls [25–27], it has been shown that Fc dectin-1 and Fc dectin-2 do not physically bind to the cryptococcal yeast cells [12,28]. Therefore, the exposure of such PAMPs is potentially regulated in cryptococcal cells. Similarly, we hypothesized that cryptococcal cells can alter the antigenic potential, including the expression and exposure of PAMPs, in response to the environmental conditions. Similar models in the previous studies have revealed that *C*. *neoformans mar1*Δ and *rim101*Δ upregulate antigenic potential in tissue culture conditions [29,30], and that *Candida albicans* masks its β-glucan based on the carbon sources of host niches [31].

In the present study, we investigated whether culture conditions influence the immunostimulatory ability of *C*. *gattii* capsular and acapsular strains. We mainly investigated the exposure patterns of dectin-1 ligands, including β-1,3-glucan, on *C*. *gattii* cells, because β-glucan is a target molecule in the diagnosis of invasive fungal diseases, although the sensitivity and specificity of β-glucan levels in serum are not adequate for the diagnosis of cryptococcosis [32]. In addition, dectin-1 was first discovered as an anti-fungal immune-receptor [10], and the findings of some recent studies suggest that dectin-1 can also recognize the endogenous ligands in mammalian cells [33,34]. Here, we demonstrate that *C*. *gattii* can expose dectin-1 and dectin-2 ligands via a capsule-independent mechanism when cultured in synthetic dextrose (SD) medium [may also be referred to as yeast nitrogen base (YNB) with glucose medium] and that PAMP-exposed *C*. *gattii* cells are more effectively recognized by DCs compared to non-exposed controls.

## Materials and methods

### Ethics

All animal experiments were approved by the ethical committee of the National Institute of Infectious Disease, Japan (approval numbers 116019, 116025, 116124, and 117032) and were performed in accordance with the approved guidelines and regulations.

### Mice

C57BL/6J mice were purchased from Japan SLC, Inc., and maintained under specific-pathogen-free conditions at the National Institute of Infectious Diseases of Japan.

### Fungi

*C*. *albicans* SC5314, *C*. *neoformans* H99, *C*. *gattii* R265, *C*. *gattii* PNG18, and derivative strains were cultivated for 2 days at 30°C in the following medium or agar plate: yeast extract–peptone–dextrose (YPD) broth [1% (w/v) yeast extract, 2% (w/v) Bacto peptone, and 2% (w/v) dextrose, premix powder purchased from BD Difco], SD medium [0.67% (w/v) YNB with amino acids and ammonium sulfate (BD Difco), and 2% (w/v) dextrose], Sabouraud broth [1% (w/v) polypeptone (BD Difco) and 2% (w/v) dextrose], and potato dextrose agar [0.4% (w/v) potato starch, 2% (w/v) dextrose, and 1.5% (w/v) agar, premix powder purchased from BD Difco] [2,5,35]. The fungal cells were also grown in SD medium containing 1% (w/v) Bacto yeast extract (BD Difco) or 25 mM neutral buffer, 4-(2-hydroxyethyl)-1-piperazineethanesulfonic acid (HEPES). Fungal cells were washed three times with phosphate-buffered saline (PBS) after cultivation.

*CAP59* (GenBank accession: CGB_A8090W) is a gene associated with capsule biosynthesis in *C*. *gattii* [36,37]. The *CAP59* deletant (CAP59Δ) and revertant (CAP59C) were derived from the parent strain, *C*. *gattii* PNG18, and they were constructed in a manner similar to the way *CAP60* deletant (CAP60Δ) and revertant (CAP60C) were constructed as described previously [5]. In CAP59Δ, the open reading frame of *CAP59* was replaced entirely by a disruption cassette containing nourseothricin-resistant gene *NAT1* via double homologous crossover, whereas the complementation vector pJAF12-CAP59 was integrated into the *CAP59* locus via a single homologous crossover to construct the revertant CAP59C. PCR was performed to confirm appropriate integration events. The primers used for the strain construction and confirmation are listed in S1 Table. Capsule formation of PNG18, CAP59Δ, and CAP59C were also confirmed using the conventional India ink method as described previously [5]. Capsule polysaccharide-specific monoclonal antibody (mAb), 18B7 (Merk Millipore, #MABF2069), bound to the cell surfaces of PNG18 and CAP59C, but not to that of CAP59Δ (S1 Fig).

A previous study indicated that heat-killed *C*. *albicans* increased β-glucan exposure on the cell surface [38]; thus, a part of fungal suspension was boiled for 1 h to prepare the heat-killed fungal cells as previously described [5].

SD medium-induced fungal cells were treated by 0.5 mg/mL Zymolyase-20T (Nacalai Tesque Inc.) in 0.1 M sodium phosphate buffer with a pH of 6.5 (BD Bioscience Cat#550536) for 1 h at 45°C to lyse β-1,3-glucan in the fungal cells. After the digestion, fungal cells were washed three times with PBS.

### Bone marrow-derived dendritic cells (BMDCs)

BMDCs were prepared as described previously [5,39]. In brief, bone marrow (BM) cells were harvested from the femurs and tibiae of C57BL/6J mice. After the lysis of erythrocytes, the BM cells were cultivated in complete Roswell Park Memorial Institute (RPMI) 1640 medium, supplemented with 10% (v/v) fetal bovine serum (FBS), 1% (v/v) streptomycin–penicillin solution, 44 μM 2-mercaptoethanol, and 10 ng/mL of mouse granulocyte-macrophage colony-stimulating factor (mGM-CSF). On day 6, non-adherent cells were collected and used as BMDCs.

### Fc fusion proteins

We cloned cDNA encoding the carbohydrate recognition domain (CRD) of mouse dectin-1 from BMDCs from C57BL/6J mice using primers pDectin1-CRD-Fw1 (5′-TTGCACTAAGTCTTGCACTTGTCACatgccttcctaattggatcatgcatg-3′) and pDectin1-CRD-Rv1 (5′-GCTTACAACCACAATCCCTGGGCACcagttccttctcacagatactgtat-3′). The pCAG-Neo mIgG1-Fc plasmid (FUJIFILM Wako Pure Chemical Corp.) was digested with XhoI–SpeI, and the cDNA fragment was inserted using the Gibson Assembly System (New England Biolabs Inc.) to construct the expression vector pmDectin-1_mIgG1-Fc. Our dectin-1 CRD cDNA sequence was identical to that of Clec7a-202, as recorded in the Ensembl database (http://www.ensembl.org/index.html, transcript ID: ENSMUST00000184581.2).

The expression vector pmDectin-1_mIgG1-Fc was transfected into HEK293T cells using PEI-MAX (Polysciences Inc.) or GenePORTER2 (Gelantis Inc.), and cells were cultivated in RPMI 1640 medium with 10% (w/v) FBS for 2 days at 37°C, 5% CO_2_ to generate Fc dectin-1 in the culture supernatant. Culture supernatant was stored at −80°C prior to use in the Fc dectin-1 deposition assay.

Fc dectin-2 was purchased from Enzo Life Science, Inc., was reconstituted with sterile water, and was stored at −80°C.

### Binding assay

For the Fc dectin-1 deposition assay, the fungal cells were treated with culture supernatant containing Fc dectin-1 for 30 mins at 37°C as described previously [38,40]. To evaluate whether Fc dectin-1 bound to β-glucan on fungal cells, the competitive soluble β-glucan, schizophyllan (SPG, final concentration 500 μg/mL; Invivogen), was added to the culture supernatant, and the mixture of SPG and Fc dectin-1 was incubated for 60 min at room temperature prior to the binding assay. Anti-β-1,3-glucan mAb (BioSupplies Australia, #400–2) was used to evaluate β-1,3-glucan exposure. Fc dectin-1 and anti-β-1,3-glucan binding to the cells were labeled using Alexa Fluor488 anti-mouse IgG (Jackson ImmunoResearch Laboratories Inc., #115-545-071). FACS buffer [PBS containing 2 mM ethylenediaminetetraacetic acid, 0.5% (w/v) bovine serum albumin, and 0.1% (w/v) sodium azide] was used for the cell treatment and washing in these experiments.

To evaluate the exposure of dectin-2 ligands, the fungal cells were treated with 2.5 μg/mL Fc dectin-2 solution for 60 mins at room temperature as described previously [12]. The deposition of Fc dectin-2 on the fungal cells was labeled with Alexa Fluor647 anti-human IgG (Jackson ImmunoResearch Laboratories Inc., #709-605-149). Hank's Balanced Salt Solution containing magnesium and calcium (Nacalai Tesque Inc.) was used for the cell treatment and washing in this experiment.

Fluorescence intensity of the labeled fungal cells were evaluated via flow cytometer and the analysis software. Fungal cells were also observed using a confocal laser-scanning microscope LSM 700 (Carl Zeiss), and the images were analyzed using ZEN software (Carl Zeiss).

### Immunoblotting

All procedures were performed as described previously [6,41]. In brief, BMDCs (1 × 10^6^ cells) and heat-inactivated fungal cells (2 × 10^6^ cells) were placed in 96-well round bottom plates containing 200 μL of the complete RPMI1640 medium supplemented with mGM-CSF. After 10 min of stimulation, BMDCs were washed with PBS and fixed for 60 mins with 10% (w/v) trichloroacetic acid. The fixed cells were treated with a lysis buffer containing urea, Triton X-100, and lithium dodecyl sulfate. The protein disulfide bonds in lysates were reduced with 1,4-dithiothreitol solution. Finally, the protein solution was neutralized using a tris(hydroxymethyl)aminomethane solution, and a neutral pH was confirmed by adding bromophenol blue solution.

PVDF Blocking Reagent (Toyobo) and Can Get Signal Immunoreaction Enhancer Solution (Toyobo) were respectively used for the blocking and antibody treatments respectively. The following antibodies were used in the experiment: Syk pY525/pY526 monoclonal antibody (1:1000 dilution, clone C87C1; Cell Signaling Technology), Akt pS473 monoclonal antibody (1:1000 dilution, clone M89-61; BD Bioscience), ERK1/2 pT202/pY204 (1:1000 dilution, clone 20A; BD Bioscience), anti-mouse α-tubulin rabbit polyclonal antibody (1:5000 dilution, 2144S; Cell Signaling Technology), anti-mouse immunoglobulin G (IgG), Fcγ fragment specific peroxidase AffiniPure (1:5000 dilution, 115–035–071; Jackson ImmunResearch), and anti-rabbit IgG (H + L) peroxidase AffiniPure (1:5000 dilution, 111-035-003; Jackson ImmunResearch).

### Phagocytosis

Fluorescent labeling of fungal cells and a phagocytosis assay were performed as described previously [6,28]. In brief, fungal cells were labeled with AlexaFluor488 NHS Ester (final 1 μg/mL, 1:10000 dilution; ThermoFisher Scientific) for 30 min at 37°C. BMDCs (4 × 10^6^ cells) and the AF488-labeled fungal cells (1.2 × 10^7^ cells) were placed in 12-well flat-bottom plates containing 1 mL of the complete RPMI1640 medium supplemented with mGM-CSF, and the suspension was centrifuged for 5 min at 320 × *g* at room temperature before being incubated for 3 h. All cells were harvested using a cell scraper and used for flow cytometry analysis.

### Flow cytometry

BMDCs were labeled with CD11b (M1/70; BioLegend) and CD11c (N418; BioLegend) antibodies after blocking Fc receptors using anti-CD16/32 mAb (clone 93; BioLegend). FACS buffer was used to stain and wash the cells. Data on the fungal cells and BMDCs were acquired using a BD FACSCalibur (BD Bioscience) and a BD FACSCanto II flow cytometer (BD Bioscience), respectively. Data were analyzed using FlowJo software (Tree Star, Inc.).

### Cytokines

BMDC cytokine production was measured as described previously [5,39,42] In brief, BMDCs (2 × 10^5^ cells/200 μL) were stimulated by heat-killed fungal cells in 96-well flat-bottom plates for 24 h. Culture supernatants were collected, and cytokine amounts were measured using enzyme-linked immunosorbent assay (ELISA). A MaxiSorp plate (Thermo Fisher Scientific), a BD OptEIA ELISA set for IL-6 (BD Bioscience), and a DuoSet ELISA kit for IL-23 (R&D Systems) were used according to the manufacturers’ instructions.

### Statistical analysis

Prism7 software (GraphPad Software, Inc.) was used for all statistical analyses. *P*-values less than 0.05 were considered statistically significant.

## Results

### Dectin-1 ligands are induced on *C*. *gattii* cells growing in SD medium

Although dectin-1 and dectin-2 generally recognize fungal cell wall components, soluble recombinant receptors, Fc dectin1 and Fc dectin-2, do not physically bind to the yeast forms of capsular or acapsular *C*. *neoformans* strains [12,20]. Based on the previous studies, we hypothesize that culture conditions influence the exposure of PAMPs in *C*. *gattii* cells.

**Fig 1:**
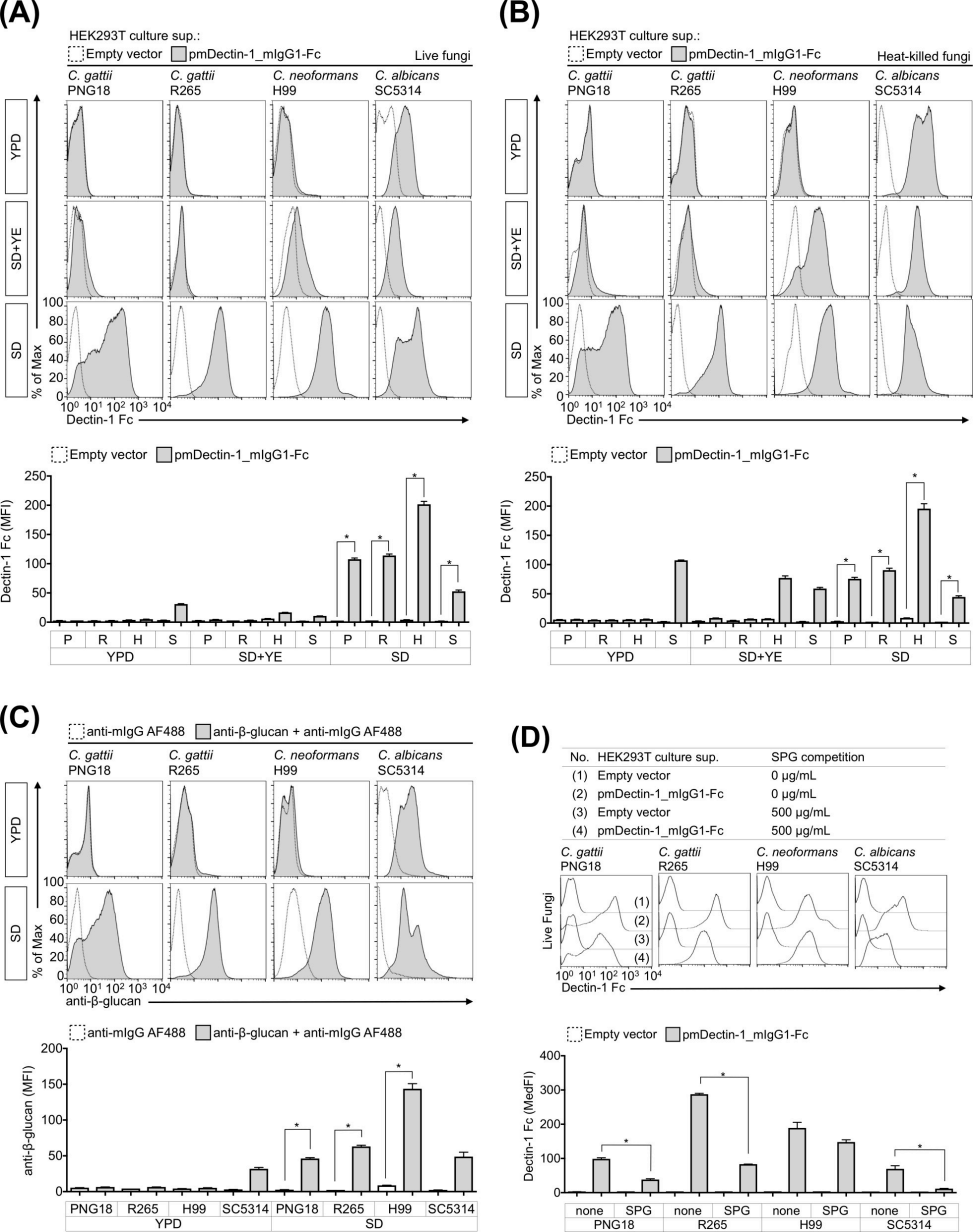
SD medium induces dectin-1 ligands on cryptococcal cells. Deposition of Fc dectin-1 was measured on live fungal cells (A and D) and heat-killed fungal cells via flow cytometry (B). In a similar manner, β-glucan exposure on heat-killed fungal cells was evaluated using anti-β-1,3-glucan mAb (C). The flow cytometry profile and bar graphs of the mean fluorescence intensity (MFI) or median fluorescence intensity (MedFI) are depicted. Representative data (mean ± SDs) from three independent experiments are shown. *: *P* < 0.05 as determined via an unpaired *t*-test, P: *C*. *gattii* PNG18, R: *C*. *gattii* R265, H: *C*. *neoformans* H99, S: *C*. *albicans* SC5314.

**Fig 2:**
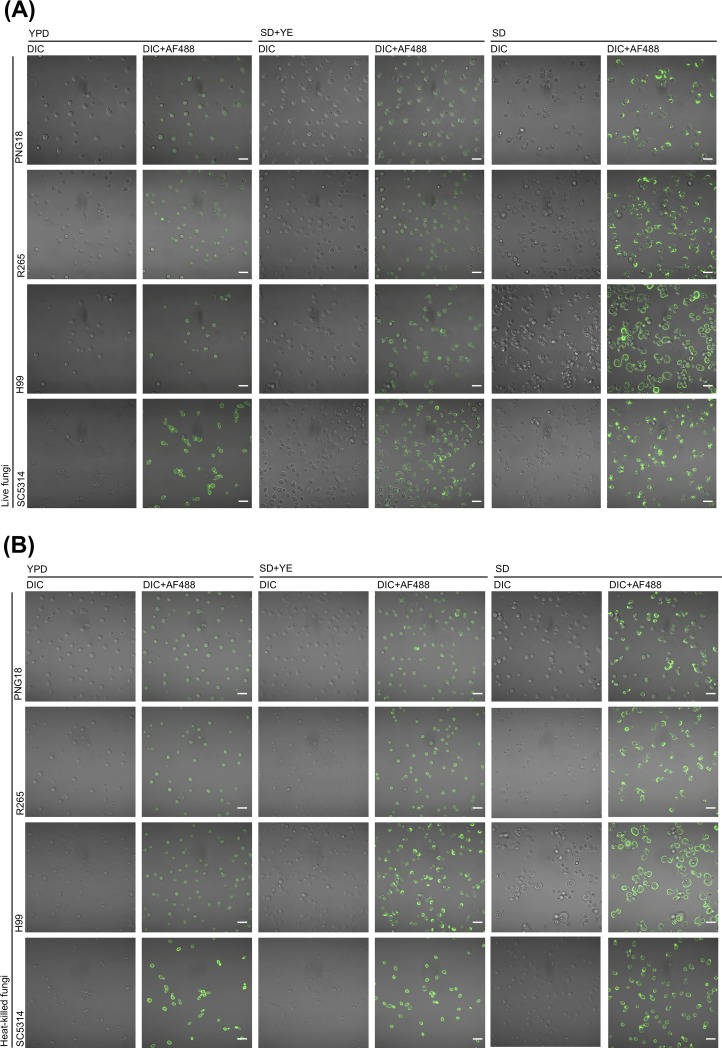
Soluble dectin-1 binds to cell surface of cryptococcal cells. Deposition of Fc dectin-1 on live fungal cells (A) and heat-killed fungal cells (B) was observed using a confocal laser-scanning microscope, and differential interference contrast (DIC) images and merge fluorescent images (DIC + AF488) are shown. Bar = 10 μm.

We first investigated the exposure of dectin-1 ligands on *C*. *gattii* growing in the common fungal culture media, compared to *C*. *neoformans* and *C*. *albicans* (Figs 1 and 2). Fc dectin-1 and anti-β-1,3-glucan mAb bound to the cell surface of live and heat-killed *C*. *albicans* cultivated in any medium (Fig 1A–1C), and the binding of Fc dectin-1 to *C*. *albicans* was decreased in the presence of competitive soluble β-glucan SPG (Fig 1D). The Fc dectin-1 did not bind to *C*. *albicans* treated by β-1,3-glucanase, Zymolyase (S2 Fig). The binding of Fc dectin-1 to the cell surface of *C*. *albicans* was also confirmed by confocal microscopy (Fig 2A and 2B). These findings are consistent with a previous study showing that the Fc dectin-1 binds to the β-glucan on fungal cells [38] and suggest that the experiment system is functioning correctly.

In contrast to *C*. *albicans*, Fc dectin-1 and anti-β-1,3-glucan mAb bound to the cell surface of *C*. *gattii* and *C*. *neoformans* cultivated in SD medium but not in YPD medium (Figs 1A–1C, 2A, and 2B). A previous study indicated that heat-killed *C*. *albicans* increased β-glucan exposure on the cell surface [38]; however, heat inactivation did not affect the exposure of dectin-1 ligands on *C*. *gattii* and *C*. *neoformans* cells growing in YPD medium (Figs 1A, 1B, 2A, and 2B). The yeast extract in SD medium negatively affected the exposure of dectin-1 ligands on *C*. *gattii* and *C*. *neoformans* cells (Figs 1A, 1B, 2A, and 2B), and the binding of Fc dectin-1 to *C*. *gattii* was significantly inhibited in the presence of SPG (Fig 1D). The deposition of Fc dectin-1 was also decreased on *C*. *gattii* treated by Zymolyase (S2 Fig). These results suggest that Fc dectin-1 binds to the dectin-1 ligands, including β-glucan, on yeast form of *C*. *gattii* cells growing in SD medium and that culture condition affect the antigenic potential of *C*. *gattii*.

### *C*. *gattii* acapsular strain CAP59Δ does not always expose dectin-1 ligands

**Fig 3:**
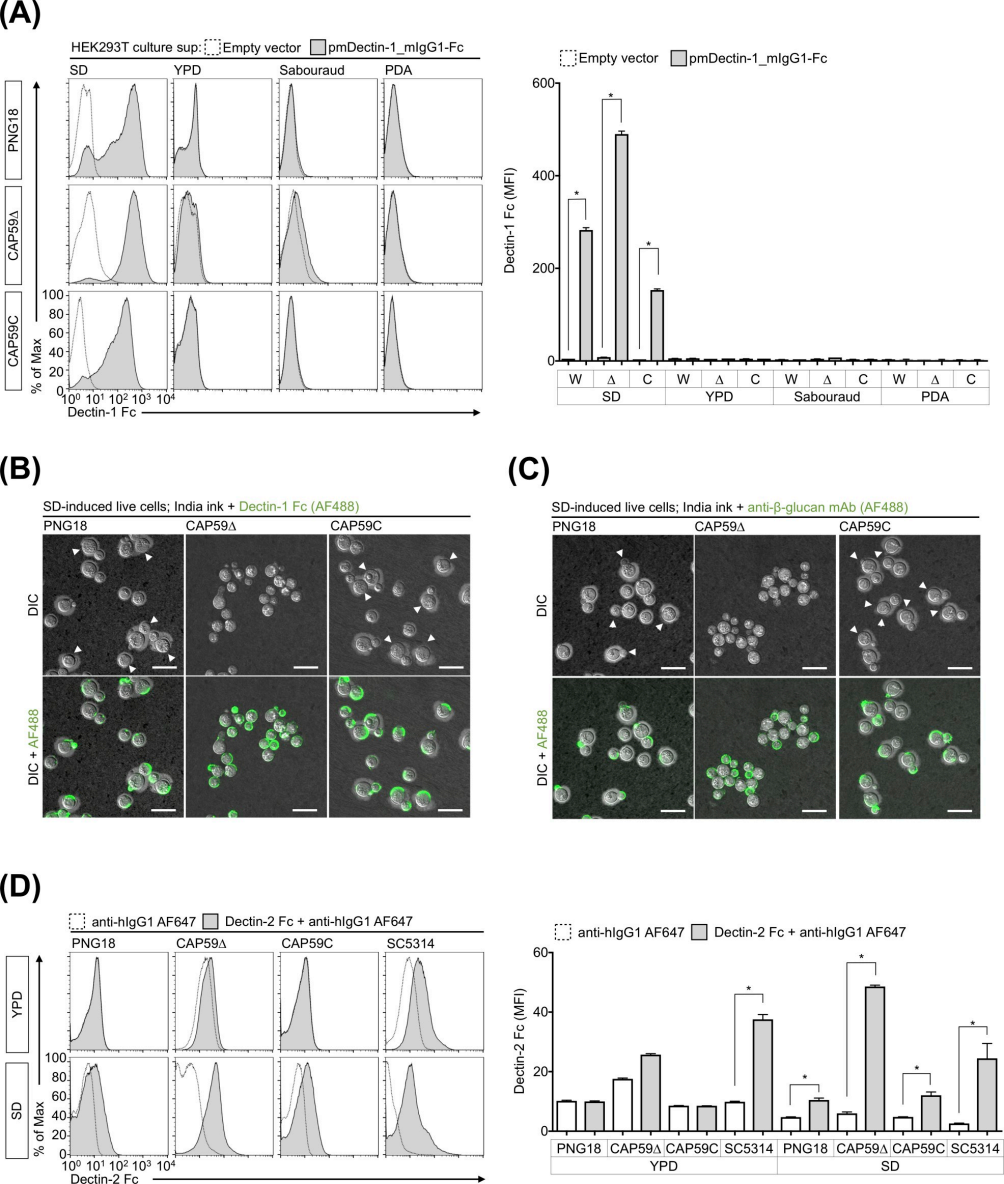
*C*. *gattii* acapsular strain CAP59Δ also requires SD medium to induce dectin-1 and dectin-2 ligands. Deposition of Fc dectin-1 (A and B), anti-β-1,3-glucan mAb (C), and Fc dectin-2 (D) on fungal cells were evaluated as described in Fig 1 and Fig 2. Live fungal cells (B and C) and heat-killed fungal cells (A and D) were used for the deposition assay. The fluorescent signal was detected by flow cytometry (A and D) and confocal laser-scanning microscopy (B and C). Capsule formation was observed using the conventional India ink method. Arrow heads indicate the capsule erosion sites on *C*. *gattii* cells. Bar = 10 μm. Representative bar graph (mean ± SDs) from three independent experiments are shown. *: *P* < 0.05 as determined via an unpaired *t*-test, W: *C*. *gattii* PNG18 (parent strain), Δ: *C*. *gattii* CAP59Δ (*CAP59* deletant, acapsular strain), C: *C*. *gattii* CAP59C (*CAP59* revertant, capsular strain).

In contrast to the capsular wild type strain, *C*. *gattii* acapsular mutant was instantly recognized and engulfed by BMDCs in the absence of opsonization [5]. The capsular components potentially masked PAMPs, including dectin-1 ligands, in cryptococcal cells. Therefore, we subsequently compared the exposure of dectin-1 ligands among *C*. *gattii* acapsular mutant CAP59Δ, the parent strain PNG18, and the revertant strain CAP59C (Fig 3A). The experiment revealed that CAP59Δ also required SD medium, not PDA, YPD, and Sabouraud medium, to induce dectin-1 ligands, and the deposition of Fc dectin-1 on CAP59Δ cells was higher than on capsular strains PNG18 and CAP59C cultured in SD medium (Fig 3A). Although it has been shown that the tissue culture media such as RPMI1640 medium containing FBS, can induce cell wall remodeling in cryptococcal cells [29], dectin-1 ligands were not strongly induced in cryptococcal cells cultivated in the tissue culture medium (S3 Fig). The findings suggest that a unique environment is required to induce PAMPs, including dectin-1 ligands, even though capsular components are deficient.

It has been previously reported that the cell walls of *C*. *neoformans* are reconstructed in SD medium via suicide autolytic activity [27]. The study also demonstrated that β-1,3-glucan and chitin contents significantly increased in *C*. *neoformans* cells cultured in SD medium as well as the erosion of capsular cell wall and aberrant cell shape in *C*. *neoformans* cells under electron and light microscopy [27] The reported abnormal cell shapes of *C*. *neoformans* were consistent with the microscopic images presented in Fig 2. We subsequently observed the binding sites of Fc dectin-1 and anti-β-1,3-glucan mAb under a confocal microscope using the conventional India ink method (Fig 3B and 3C). It revealed that Fc dectin-1 and anti-β-1,3-glucan mAb bound to the erosion sites in the capsules of PNG18 and CAP59C cells, while they were uniformly exposed on the surfaces of CAP59Δ cells (Fig 3B and 3C). In addition, Fc dectin-2 bound to *C*. *gattii* cells cultured in SD medium but not to that in YPD medium, and the deposition of Fc dectin-2 on CAP59Δ cells was higher than in capsular strains, including PNG18 and CAP59C (Fig 3D). The results suggest that cell wall remodeling in SD medium is likely associated with the alteration of antigenic potential in *C*. *gattii*.

### Acidification of SD medium is a key factor inducing dectin-1 ligands on *C*. *gattii*

**Fig 4:**
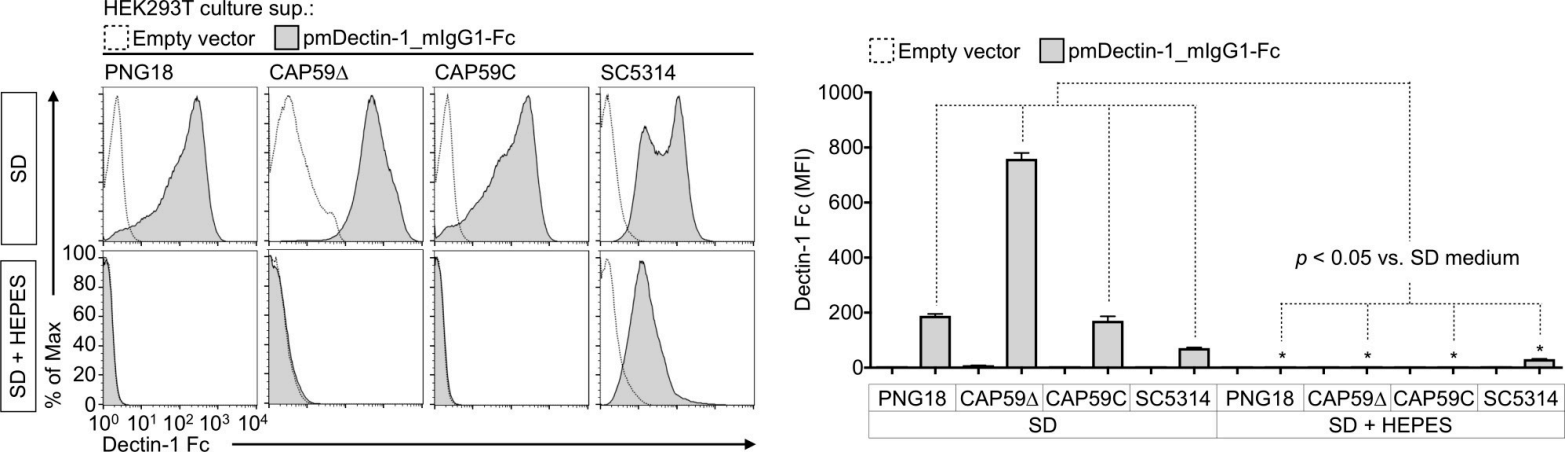
HEPES-buffered SD medium does not induce dectin-1 ligands on *C*. *gattii*. Deposition of Fc decin-1 on live fungal cells were evaluated via flow cytometry as described above. Representative bar graph (mean ± SDs) from three independent experiments are shown. *: *P* < 0.05 versus counterparts of SD medium without HEPES using an unpaired *t*-test.

A previous study reported that the acidification of SD medium is a key signal inducing cell wall remodeling in *C*. *neoformans* [27]. We hypothesized that the acidification of SD medium was also required for antigenic alteration, including the exposure of dectin-1 ligands on *C*. *gattii*. Therefore, we tested whether HEPES-buffered SD medium (SD + HEPES) minimized the exposure of dectin-1 ligands on *C*. *gattii* (Fig 4). The initial pH of the SD medium was approximately 5.0, whereas after 2 days of *C*. *gattii* and *C*. *neoformans* culture, the pH of the SD medium was 1.9–2.1. The pH values of the before and after the culture in SD + HEPES medium was in the range 7.0–7.3. Consequently, Fc dectin-1 bound to *C*. *albicans* cells cultured in SD + HEPES medium but not to *C*. *gattii* PNG18, CAP59Δ, and CAP59C strains (Fig 4). SD + HEPES medium-induced cryptococcal cells exhibited a darker fluorescent signal than the SD medium-induced controls after chitin and chitooligomer staining with calcofluor white (S4 Fig). Additionally, we tested whether dectin-1 ligands in cryptococcal cells were reversibly suppressed in SD + HEPES medium after induction in SD medium (S5 Fig). This test revealed that the exposure of dectin-1 ligands was completely suppressed again in cryptococcal cells that were sequentially cultivated in SD + HEPES medium after induction in SD medium (S5 Fig). The findings suggest that acidic environments are likely to alter the antigenic potential of *C*. *gattii*.

### BMDCs were strongly activated by *C*. *gattii* cells cultured in SD medium

**Fig 5:**
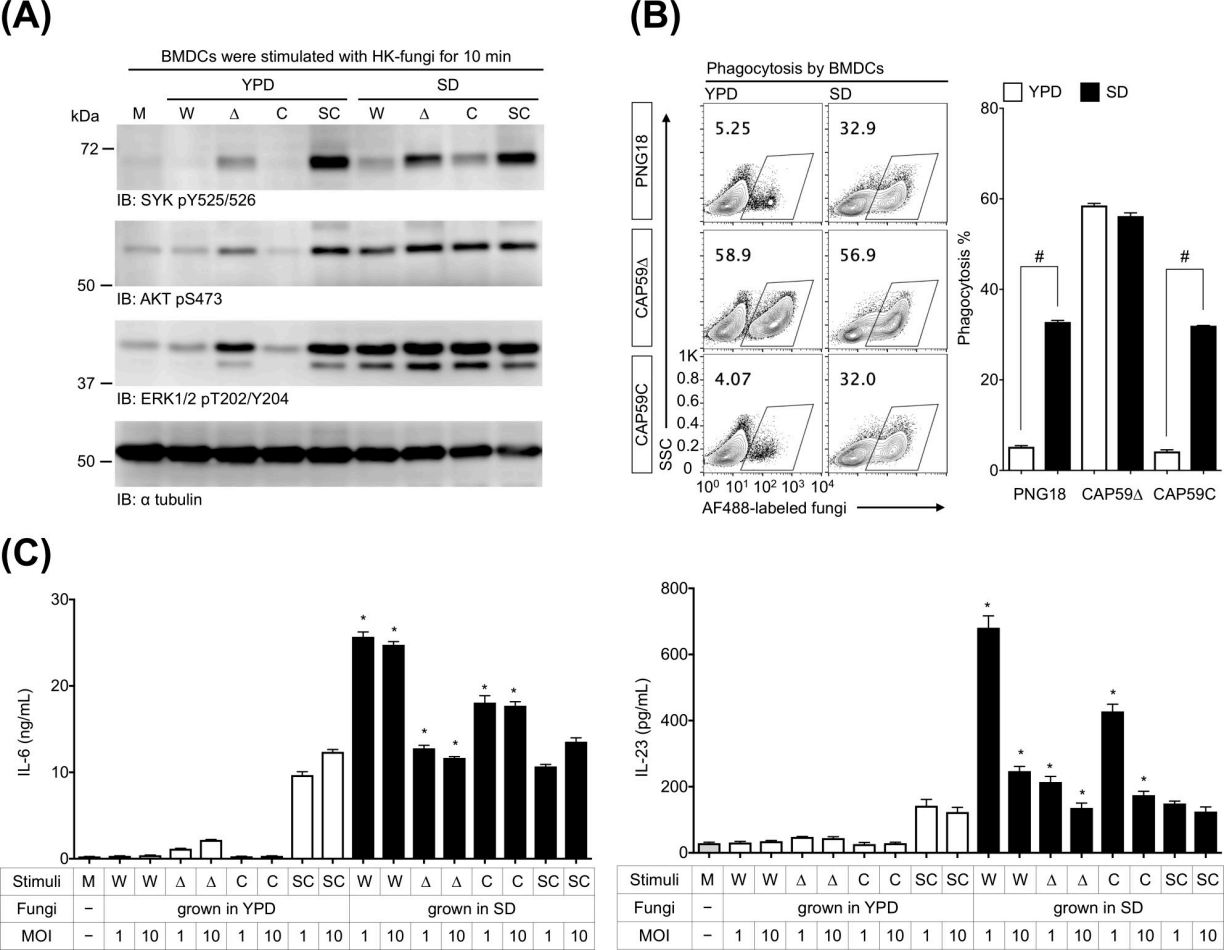
BMDCs were strongly activated by *C*. *gattii* cells growing in SD medium. BMDCs were incubated with heat-killed (HK) fungal cells for 10 min (A, MOI = 2), 3 h (B, MOI = 3), or 24 h (C). Protein phosphorylation (A), phagocytosis (B), and cytokine production (C) were evaluated by Western blotting, flow cytometry, and ELISA, respectively. For flow cytometry analysis, gates were set for CD11b^+^ CD11c^+^ BMDCs. Representative bar graph (mean ± SDs) from three independent experiments are shown. #: *P* < 0.05 as determined via an unpaired *t*-test, *: *P* < 0.05 versus counterparts of YPD medium using an unpaired *t*-test, W: *C*. *gattii* PNG18, Δ: *C*. *gattii* CAP59Δ, C: *C*. *gattii* CAP59C, SC: *C*. *albicans* SC5314.

Dectin-1 and dectin-2 ligands were exposed on *C*. *gattii* PNG18, CAP59Δ, and CAP59C strains cultured in SD medium, but not in most common fungal culture medium YPD medium (Fig 3). Therefore, we subsequently evaluated the actual immunostimulatory ability of *C*. *gattii* PNG18, CAP59Δ, and CAP59C cultured in SD medium (Fig 5). In the experiment, we used BMDCs to analyze innate immune responses to SD medium-induced *C*. *gattii* cells as described in a previous study [5] because BMDCs express dectin-1 and dectin-2 [16,22].

It has been demonstrated that phospho-Syk, -Akt, and -Erk1/2 increase in BMDCs after 10–30 min-stimulation with *C*. *albicans* [43,44], and our experiment revealed that both YPD and SD medium-induced *C*. *albicans* cells similarly induced phosphorylation in BMDCs for 10 min (Fig 5A). The phosphorylation did not increase in BMDCs stimulated with *C*. *gattii* capsular strains PNG18 and CAP59C growing in YPD, whereas SD medium-induced *C*. *gattii* strains, CAP59Δ particularly, increased the phosphorylation in BMDCs greatly (Fig 5A). The results suggest that BMDCs instantly recognize capsular *C*. *gattii* cultured in SD medium.

We have previously demonstrated that BMDCs and neutrophils do not phagocytize unopsonized capsular cryptococcal cells cultured in YPD [5,6]. Therefore, we tested whether BMDCs phagocytize capsular *C*. *gattii* cells cultured in SD medium (Fig 5B). The experiment revealed that BMDCs more efficiently engulfed SD medium-induced PNG18 and CAP59C than their YPD-induced control counterparts (Fig 5B). In contrast to capsular strains, both YPD and SD medium-induced acapsular CAP59Δ strains were comparably engulfed by BMDCs (Fig 5B).

In general, BMDCs secrete substantial amounts of inflammatory cytokines after fungal recognition and phagocytosis [14]. Therefore, we measured the amounts of IL-6 and IL-23 in culture supernatants of BMDCs stimulated by *C*. *gattii* and *C*. *albicans* cultured in YPD and SD (Fig 5C). BMDCs produced IL-6 and IL-23 after stimulation with *C*. *albicans* regardless of culture conditions, whereas SD medium-induced *C*. *gattii* strains induced significantly greater production of IL-6 and IL-23 by BMDCs than YPD medium-induced *C*. *gattii* strains (Fig 5C). Despite the fact that SD and YPD medium-induced CAP59Δ were comparably engulfed by BMDCs (Fig 5B), SD medium-induced CAP59Δ more effectively induced the protein phosphorylation (Fig 5A) and the production of inflammatory cytokines (Fig 5C). Overall, the findings demonstrate that immunostimulatory potential in *C*. *gattii* capsular and acapsular strains is upregulated in SD medium.

## Discussion

In the present study, we demonstrated that dectin-1 and dectin-2 ligands were exposed on *C gattii* cells growing in SD medium and that SD medium-induced *C*. *gattii* influence the immunostimulatory activity to BMDCs.

Our recent study showed that DC-based vaccination induced lung resident memory Th17 cells, which influence the protective effects against highly virulent *C*. *gattii* infection [39]. In the DC vaccine, YPD medium-induced CAP60Δ was used as a vaccine antigen to prime BMDCs. We believe that SD medium-induced acapsular cells including CAP59Δcould be the more favorable vaccine antigens for the stimulation of protective T cells against highly virulent cryptococcosis.

It has been demonstrated that β-1,3-glucan and chitin are increased in cell walls reconstructed in *C*. *neoformans* cultured in SD medium [27]. The study also indicated that the suicide autolysis activity generated dead cells in SD medium [27]. In the present study, the viability of *C*. *gattii* cells was maintained in SD medium, compared to that of *C*. *neoformans* (S4 Fig). In addition, we observed the aberrant cell shapes of *C*. *neoformans* cultured in SD medium, whereas only partial erosion of the capsule was observed in *C*. *gattii* cells (Figs 2–3, and S4 Fig). Although the autolysis activity in SD medium has also been observed in the model fission yeast, *Schizosaccharomyces pombe* [45], the physiological role of cryptococcal suicide autolysis in SD medium has remained unknown. Our findings suggest that *C*. *gattii* may have a different suicide autolysis activity in SD medium compared with *C*. *neoformans*.

SD medium-induced cell wall reconstitution or secondary cell wall formation has been perceived as a rescue system for the suicide autolysis in SD medium [27]. A previous study showed that suicide autolysis was suppressed in *C*. *neoformans* growing in SD medium supplemented with neutral buffer 3-(N-morpholino)propanesulfonic acid [27]. The finding suggested that acidification of SD medium was a key factor inducing autolysis of *C*. *neoformans* [27]. In the present study, we observed the exposure of dectin-1 ligands in the partial erosion sites of capsules on *C*. *gattii* cells growing in SD medium. Moreover, the exposure was not observed in *C*. *gattii* cells growing in HEPES-buffered neutral SD medium. In the HEPES-buffered SD medium, both *C*. *gattii* and *C*. *neoformans* had normal capsule and cell shapes (S4 Fig). The results suggest that the acidification of SD medium is a key trigger for the exposure of dectin-1 ligands. Overall, the findings suggest that cell wall remodeling may be required for the exposure of CLR ligands in cryptococcal cells.

However, we assume an additional key factor rather than medium acidification, because medium acidification was not in perfect correlation with the exposure of dectin-1 ligands. In the present study, we observed acidification in SD + YE (pH = 2.3–2.4) and Sabouraud medium (pH = 3.5–3.7) after 2-days cultivation of *C*. *gattii* cells; however, the exposure of dectin-1 ligands was not observed in *C*. *gattii* cells growing in the media (Figs 1–3). The results indicate that crude components, such as yeast extract and polypepton, in the media may suppress suicide autolysis activity and cell wall remodeling, even under acidic environments. In the case of *S*. *pombe*, autolysis is suppressed in SD medium supplemented with asparagine and phosphate [45]. The autolysis activity in *S*. *pombe* has also been observed in a *ura4* deletant growing in YPD medium, in which they cannot synthesize uridine monophosphate [46,47]; therefore, a key factor in addition to medium acidification may influence suicide autolysis and cell wall remodeling in cryptococcal cells. More studies are required to elucidate the additional signal that induces the autolysis and cell wall remodeling in *C*. *gattii* cells.

Cell wall remodeling also occurred in *C*. *neoformans* recombinant, in which the gene for pH-responsive transcriptional factor Rim101p was conserved in the fungal species [30,48]. In the cell wall of *rim101*Δ, chitin oligomer and α-glucan are significantly increased compared to that in the wild type strain. Notably, the *rim101*Δ strongly induces inflammatory responses *in vitro* and *in vivo* [30]. These results indicate that *C*. *neoformans* requires the conserved transcriptional factor for the cell wall reconstruction to evade immune surveillance [30]. Because our findings on antigenic alteration induced in SD medium are consistent with the reports associated with the *rim101*Δ phenotype, Rim101p and the relevant pathway are also potentially associated with the cell wall remodeling of *C*. *gattii* cultured in SD medium.

We speculate that the acidification of SD medium resembles the phagosome acidic environment and that CLR ligands are exposed in phagosomes. It has been demonstrated that dectin-1 and its adaptor protein CARD9 are accumulated in phagosomes [49–52]. The finding is consistent with our speculation. Whereas *C*. *gattii* generally is generally less likely to be recognized by the immune system, *C*. *gattii* cells are engulfed by innate immune cells, such as neutrophils, in the presence of fresh serum components [6]. An acidic phagosome may stimulate *C*. *gattii* cells to expose CLR ligands on the cell surface, and the exposure could enhance innate immune responses, including the production of cytokines and reactive oxygen species. Further studies are required to elucidate the physiological significance and regulation system of the antigenic alteration in acidic environments in *C*. *gattii*.

The present study had certain limitations: (1) Our findings do not fully address the relationship between the absolute amounts and the exposure levels of β-1.3-glucan and α-mannan in *C*. *gattii* cells growing in SD medium. A previous study demonstrated that the absolute amounts of β-1.3-glucan were increased in SD medium-induced cryptococcal cells, which reconstructed the cell wall via suicide autolysis activity [27]. We observed the localization of dectin-1 ligands localized on the capsule erosion sites on *C*. *gattii* cells (Fig 3B and 3C). Therefore, we presume that both amounts and exposure of β-1.3-glucan are increased in SD medium-induced *C*. *gattii* cells. (2) Our data do not address the non-glucan ligands that are recognized by dectin-1 in cryptococcal cells. The presence of SPG hardly disturbs the deposition of Fc dectin-1 on *C*. *neoforomans* H99 growing in SD medium compared with that on *C*. *gattii* cells (Fig 1D). The deposition of Fc dectin-1 was significantly decreased in *C*. *gattii* cells treated with β-1,3-glucanase; however, Fc dectin-1 still bound to *C*. *gattii* cells, even when they were treated with β-1,3-glucanase (S2 Fig). These data suggest that dectin-1 can recognize not only glucan but also other antigens expressed in cryptococcal cells. (3) Our work did not fully identify the biochemical and physiological factors affecting the exposure of PAMPs in cryptococcal cells, as described above. (4) Our data do not adequately demonstrate that the upregulation of immunostimulatory ability in SD medium-induced *C*. *gattii* cells depends on the increment of accessible CLR ligands on the cell surface. It is likely that SD medium-induced *C*. *gattii* cells increase not only the CLR ligands but also the PAMPs recognized by TLRs, NLRs, and RLRs.

In conclusion, we demonstrated that *C*. *gattii* alters its antigenic potential in response to the environment. The findings may offer insights that could facilitate the design of vaccine antigens and innate immune responses against cryptococcal cells via CLRs.

## Supporting information

S1 Table Primers used for the strain construction. (PDF)

S1 Fig Glucuronoxylomannan (GXM) production in *C*. *gattii* growing in YPD and SD medium. *C*. *gattii* PNG18, CAP59Δ, and CAP59C were labeled with anti-GXM mAb (clone 18B7). *C*. *albicans* SC5314 was used as a negative control. The fluorescent signal was evaluated by confocal laser-scanning microscopy (A) and flow cytometry (B). To verify the specific binding of 18B7 to the capsule, capsule formation was observed using the conventional India Ink method, and chitin and chitooligomers in the cell walls were stained with the fluorescent reagent calcofluor white (CFW; 10-fold dilution). The flow cytometry profile and bar graph (mean ± SDs) of MedFI are depicted. Representative data from three independent experiments are shown.
(PDF)

S2 Fig Effect of β-1,3-glucanase treatment on the deposition of Fc dectin-1. Heat-inactivated *C*. *gattii* CAP59Δ and *C*. *albicans* SC5314 were treated with Zymolyase as described in the Materials and Methods section. *C*. *albicans* SC5314 was used as a positive control. The flow cytometry profile and bar graph (mean ± SDs) of MedFI are depicted. Representative data from three independent experiments are shown. *: *P* < 0.05 as determined via an unpaired *t*-test with Welch’s correction.
(PDF)

S3 Fig The tissue culture medium is not suitable for inducing dectin-1 ligands in cryptococcal cells. *C*. *gattii* R265 and *C*. *neoformans* H99 growing in RPMI1640 medium (Nacalai 06261–65, with L-glutamine, without phenol red) with 10% FBS for 2 days under 5% CO2 at 37°C were heat-inactivated. The deposition of Fc dectin-1 on fungal cells was measured using flow cytometry. The flow cytometry profile and bar graph (mean ± SDs) of MedFI are depicted. Representative data from two independent experiments are shown.
(PDF)

S4 Fig Cell morphology, viability, and chitin contents of cryptococcal cells growing in SD and SD + HEPES medium. *C*. *gattii* PNG18 and *C*. *neoformans* H99 were cultivated in SD and SD + HEPES medium for 2 days as described in Fig 4. Capsule formation and cell morphology were observed using the conventional India Ink method (A). To evaluate cell viability, fungal cells were stained with propidium iodide (BioLegend, 1:100 dilution) for 10 min (B). Fungal suspension was diluted and spread onto YPD plates followed by overnight incubation at 30°C to determine colony forming units, CFU (B). Fungal cells were stained with calcofluor white (1:10 dilution) for 10 min to evaluate the amount of chitin and chitooligomer (C). The fluorescent signal was measured via flow cytometry (B, C). The flow cytometry profile and bar graph (mean ± SDs) and are depicted. Representative data from three independent experiments are shown. *: *P* < 0.05 as determined via an unpaired *t*-test, #: *P* < 0.05 as determined via an unpaired *t*-test with Welch’s correction. †: *P* < 0.05 versus counterparts of SD + HEPES medium as determined via an unpaired *t*-test.
(PDF)

S5 Fig Reversible induction of dectin-1 ligands in cryptococcal cells. *C*. *gattii* PNG18 and *C*. *neoformans* H99 were cultivated in SD medium for 2 days to induce exposure of dectin-1 ligands. After washing the fungal cells, fungal cells were reinoculated at 100-fold dilution in the second medium YPD, SD + HEPES, or SD medium. After 3 days of sequential cultivation, fungal cells were harvested and heat-inactivated. The deposition of Fc dectin-1 on fungal cells was evaluated as described above. The flow cytometry profile and bar graph (mean ± SDs) and are depicted. Representative data from three independent experiments are shown.
(PDF)
