# Supplementary material for: Cryptococcus gattii alters immunostimulatory potential in response to the environment
Source: PLoS One. 2019 Aug 9;14(8):e0220989. doi: 10.1371/journal.pone.0220989 (PMC6688814; doi:10.1371/journal.pone.0220989)
Supplement: S1 Table — (PDF) [file pone.0220989.s001.pdf]

S1 Table Primers used for the strain constructions

Primer	Sequence
NAT-F	AGCGGATAACAATTTCACACAGGA
NAT-R	CGCCAGGGTTTTCCCAGTCACGAC
NATcheck-F1	GTCACCAACGTCAACGCACC
cap59-upF	GTAGTTTGGTGTGGCTGTGG
cap59-upR	ACAACGTCGTGACTGGGAAAACCCTGGCGCGGGGGAGTGTTGGGGGGAG
cap59-downF	CTGTTTCCTGTGTGAAATTGTTATCCGCTGGGTTGAGAGTAGAAATTGG
cap59-downR	AAAATGTTCCCTCGCCCCGC
cap59-upF1	TCGTTGTGTGGACTTTTCAC
cap59-downR1	GCCCGAAAACAGATTTAAAC
cap59down-SacII-R	AAAACCGCGGAAAATGTTCCCTCGCCCCGC
cap59up-NotI-F	AAAAGCGGCCGCAGTTTGGTGTGGCTGTGG
Cap59rt-F	GTGCTATGCATCGCAACGCA
Cap59rt-R	ATGATCTGGGAGCGCTGCAG
