# Supplementary figures and images for: Cryptococcus gattii alters immunostimulatory potential in response to the environment
Source: PLoS One. 2019 Aug 9;14(8):e0220989. doi: 10.1371/journal.pone.0220989 (PMC6688814; doi:10.1371/journal.pone.0220989)

(A)

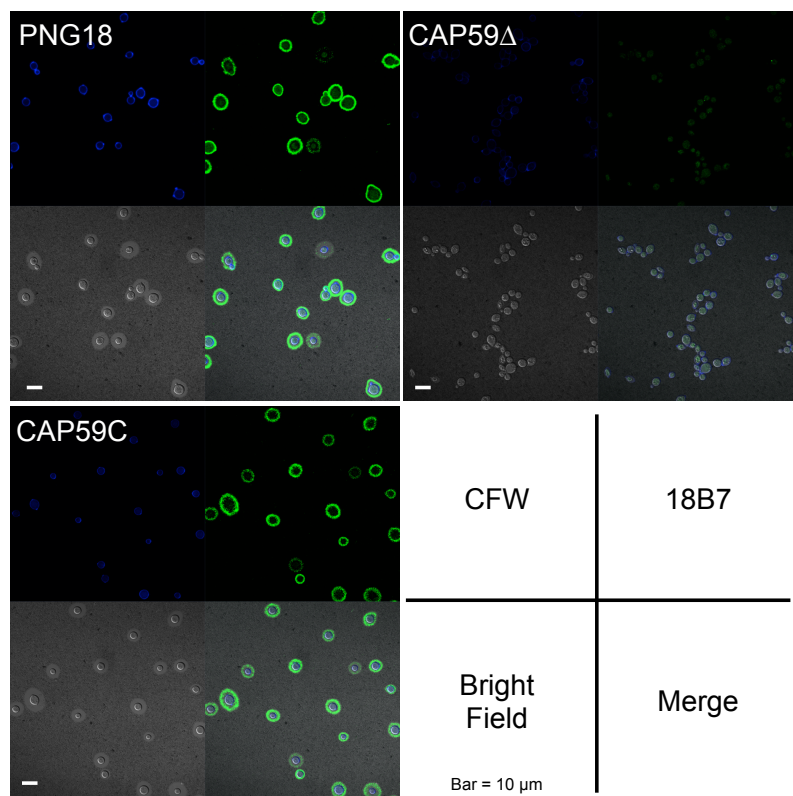

(B)

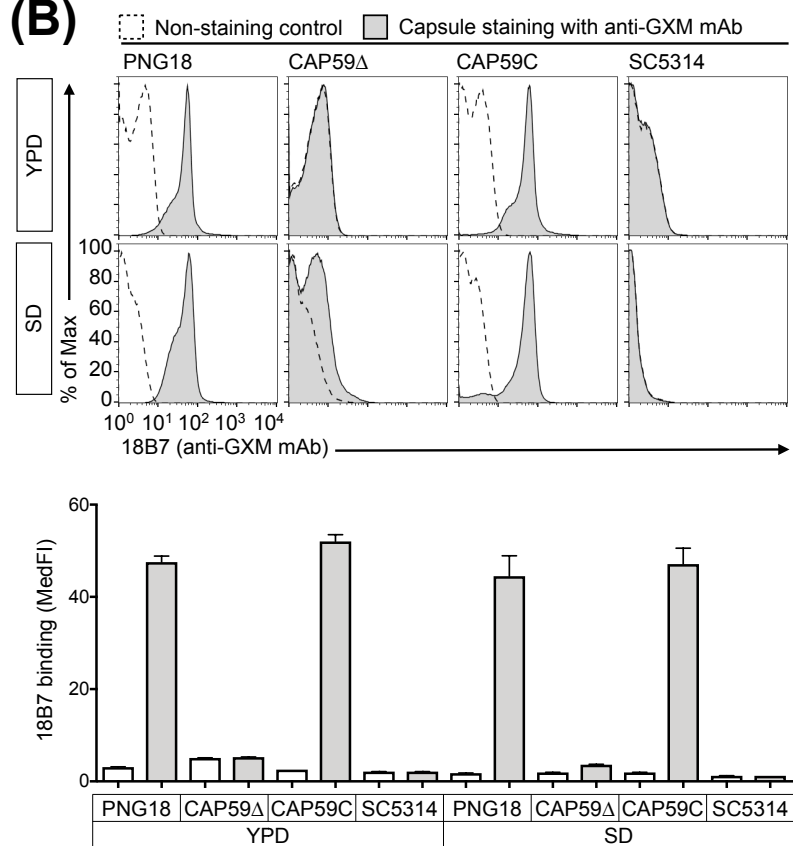

S1 Fig

Supplement: S1 Fig — C. gattii PNG18, CAP59Δ, and CAP59C were labeled with anti-GXM mAb (clone 18B7). C. albicans SC5314 was used as a negative control. The fluorescent signal was evaluated by confocal laser-scanning microscopy (A) and flow cytometry (B). To verify the specific binding of 18B7 to the capsule, capsule formation was observed using the conventional India Ink method, and chitin and chitooligomers in the cell walls were stained with the fluorescent reagent calcofluor white (CFW; 10-fold dilution). The flow cytometry profile and bar graph (mean ± SDs) of MedFI are depicted. Representative data from three independent experiments are shown. (PDF) [file pone.0220989.s002.pdf]

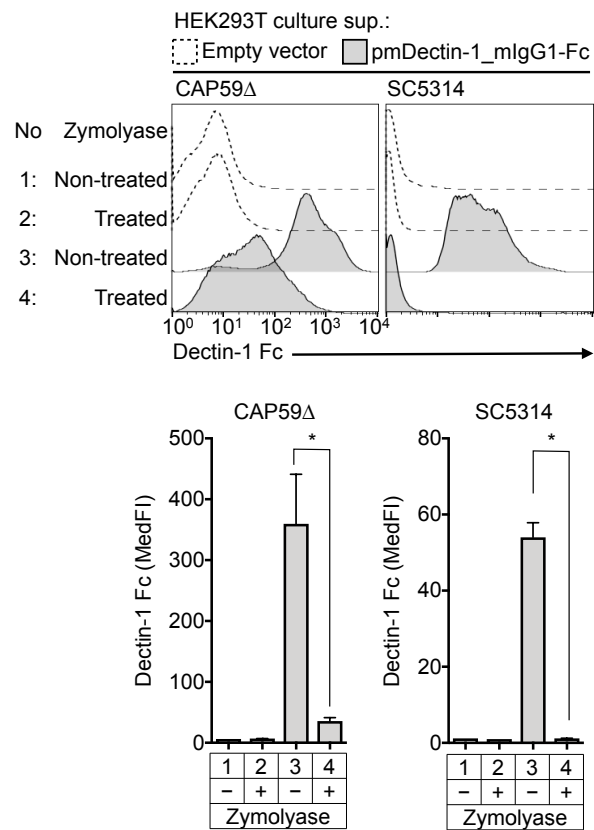

S2 Fig

Supplement: S2 Fig — Heat-inactivated C. gattii CAP59Δ and C. albicans SC5314 were treated with Zymolyase as described in the Materials and Methods section. C. albicans SC5314 was used as a positive control. The flow cytometry profile and bar graph (mean ± SDs) of MedFI are depicted. Representative data from three independent experiments are shown. *: P < 0.05 as determined via an unpaired t-test with Welch’s correction. (PDF) [file pone.0220989.s003.pdf]

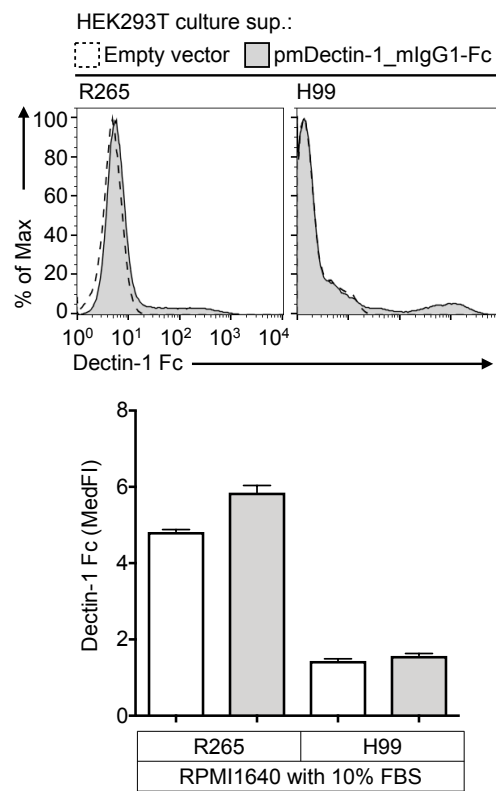

S3 Fig

Supplement: S3 Fig — C. gattii R265 and C. neoformans H99 growing in RPMI1640 medium (Nacalai 06261–65, with L-glutamine, without phenol red) with 10% FBS for 2 days under 5% CO2 at 37°C were heat-inactivated. The deposition of Fc dectin-1 on fungal cells was measured using flow cytometry. The flow cytometry profile and bar graph (mean ± SDs) of MedFI are depicted. Representative data from two independent experiments are shown. (PDF) [file pone.0220989.s004.pdf]

**(A)**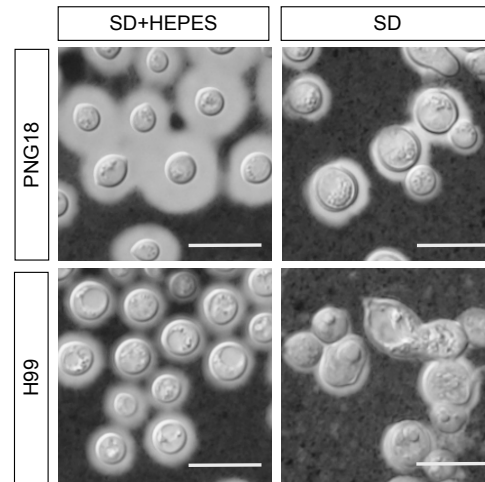**(B)**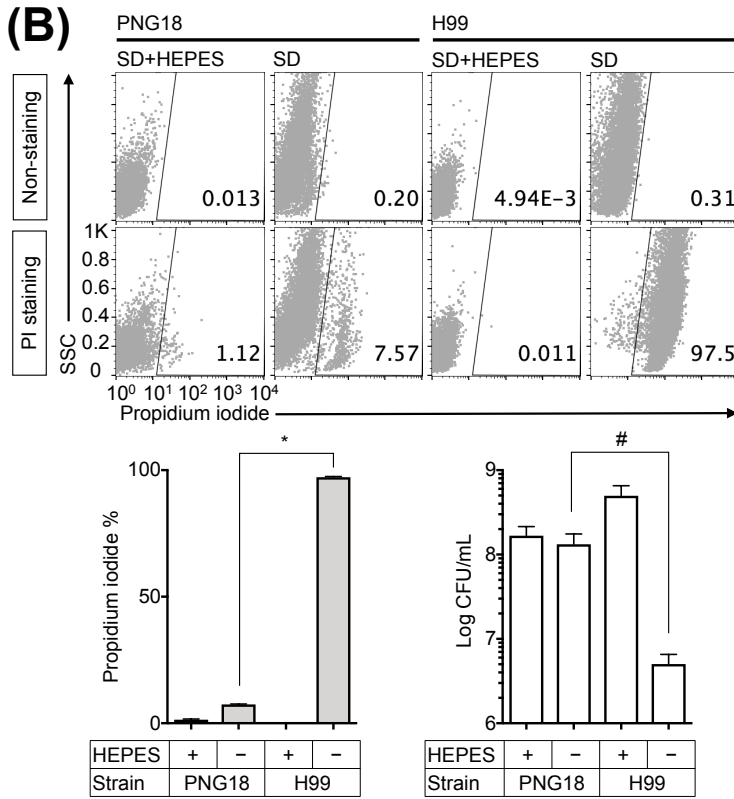**(C)**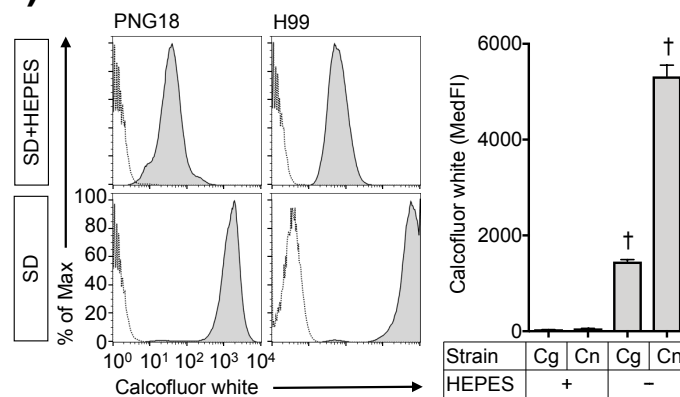

S4 Fig

Supplement: S4 Fig — C. gattii PNG18 and C. neoformans H99 were cultivated in SD and SD + HEPES medium for 2 days as described in Fig 4. Capsule formation and cell morphology were observed using the conventional India Ink method (A). To evaluate cell viability, fungal cells were stained with propidium iodide (BioLegend, 1:100 dilution) for 10 min (B). Fungal suspension was diluted and spread onto YPD plates followed by overnight incubation at 30°C to determine colony forming units, CFU (B). Fungal cells were stained with calcofluor white (1:10 dilution) for 10 min to evaluate the amount of chitin and chitooligomer (C). The fluorescent signal was measured via flow cytometry (B, C). The flow cytometry profile and bar graph (mean ± SDs) and are depicted. Representative data from three independent experiments are shown. *: P < 0.05 as determined via an unpaired t-test, #: P < 0.05 as determined via an unpaired t-test with Welch’s correction. †: P < 0.05 versus counterparts of SD + HEPES medium as determined via an unpaired t-test. (PDF) [file pone.0220989.s005.pdf]

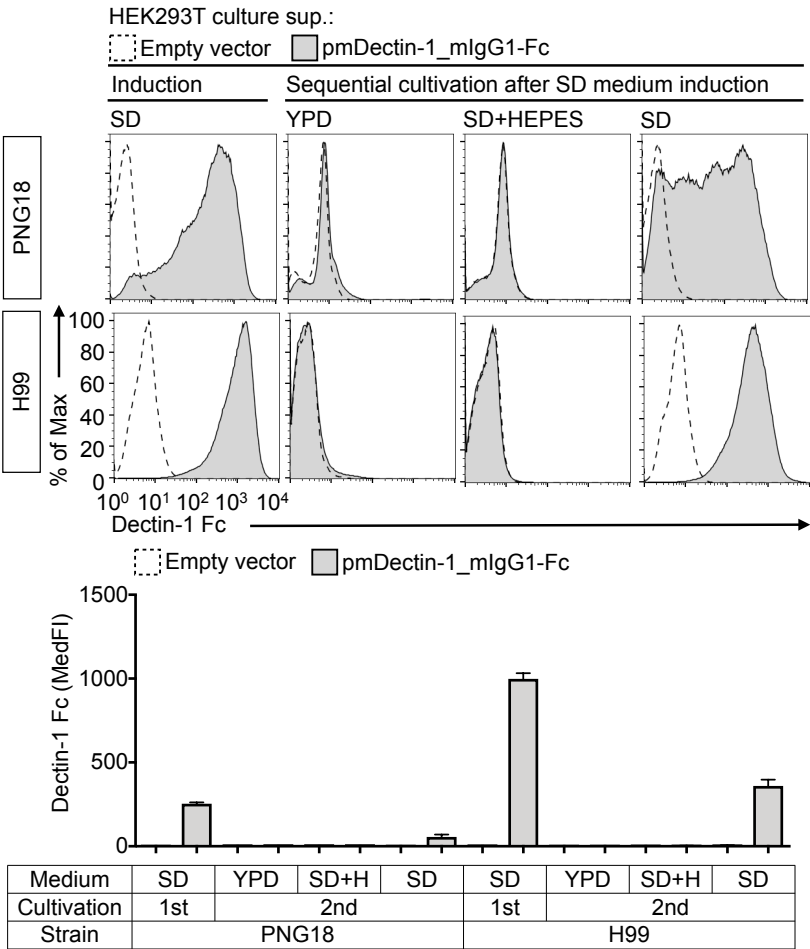

S5 Fig

Supplement: S5 Fig — C. gattii PNG18 and C. neoformans H99 were cultivated in SD medium for 2 days to induce exposure of dectin-1 ligands. After washing the fungal cells, fungal cells were reinoculated at 100-fold dilution in the second medium YPD, SD + HEPES, or SD medium. After 3 days of sequential cultivation, fungal cells were harvested and heat-inactivated. The deposition of Fc dectin-1 on fungal cells was evaluated as described above. The flow cytometry profile and bar graph (mean ± SDs) and are depicted. Representative data from three independent experiments are shown. (PDF) [file pone.0220989.s006.pdf]
